# One step conjugation of some chemotherapeutic drugs to the biologically produced gold nanoparticles and assessment of their anticancer effects

**DOI:** 10.1038/s41598-019-46602-0

**Published:** 2019-07-15

**Authors:** Behrooz Yahyaei, Parastoo Pourali

**Affiliations:** 1 Department of Medical Sciences, Shahrood Branch, Islamic Azad University, Shahrood, Iran; 2 Biological Nanoparticles in Medicine Research Center, Shahrood Branch, Islamic Azad University, Shahrood, Iran

**Keywords:** Nanoparticles, Nanoparticles, Nanoparticles, Drug development, Drug development

## Abstract

Recent research tried to analyze the conjugation of some chemotherapeutic drugs to the biologically produced gold nanoparticles (GNPs) in one step, without the use of any additional linkers. GNPs was produced using *Fusarium oxysporum* and their presence was confirmed using spectrophotometer, transmission electron microscope (TEM), X-ray diffraction (XRD) and fourier transform infrared (FTIR) analyses. In order to carry out the conjugation study, capecitabine, tamoxifen, and paclitaxel were added dropwise to the GNPs solution under stirring condition and spectrophotometer, dynamic light scattering (DLS) and FTIR analyses were performed to prove the successful conjugation. Finally, AGS and MCF7 cell lines were used for methyl thiazol tetrazolium (MTT) assay to determine the toxicity of each drug and its conjugated form. Results showed that the spherical and hexagonal GNPs with maximum absorbance peak around 524 nm and average sizes less than 20 nm were produced. FTIR analysis clarified the presence of proteins on the surfaces of the GNPs. After the conjugation process although the FTIR analysis demonstrated that all the drugs were successfully conjugated to GNPs, MTT assay revealed that unlike the paclitaxel conjugated GNPs, capecitabine and tamoxifen conjugates displayed no toxic effects due to their deactivation and low half-lives. Moreover the average size and polydispersity index (PDI) of the GNPs after conjugation with all the three tested drugs increased. In conclusion different types of drugs could conjugate to the GNPs but it is important to employ high stable forms of the drugs in the conjugation procedure.

## Introduction

Radio, chemo, and hormone therapies plus surgery are the main strategies for the treatment of different types of cancers. This multi therapy strategy is usually employed in order to increase the patient’s life. Unlike the use of different treatment strategies, many people around the world are suffered from cancer and are died yearly^1^. A successful treatment depends on the stage of the cancer and the response of the cancerous cells to the used strategies. Unfortunately, cancer drug resistance is one of the most critical problems in the cancer treatment which results in the negative prognoses^2^.

Capecitabine, tamoxifen and paclitaxel are used for the treatment of different cancer types and induce high cytotoxicity to the different cancerous cell types^3^. These drugs, like the many other ones have undesirable influences and toxic side effects on the normal cells^4^. Due to the side effects of the chemotherapeutic drugs, different drug delivery agents have been introduced and developed. Among them are Liposomes, dendrimers, nanoparticles, and nanotubes that are used as drug carriers^5^.

Sometimes, these drug delivery vehicles are designed to be selectively attached to the cancerous cells. For this aim, some especial ligands have been conjugated to the surfaces of the drug delivery vehicles so that the drug is delivered through ligands and the specific receptors of the cancerous cells^6^. Although this technique is very precise and attractive, there are some limitations such as the fact that the synthesis of the above mentioned materials is time consuming and expensive. Therefore, the alternative drug delivery systems are under consideration that chemotherapeutic conjugated nanoparticles are among them. Drugs conjugated nanoparticles have higher half lives in the body than the non-conjugated forms and due to lower lymph drainage and higher blood circulation in the tumor site, the drug loaded nanoaprticles are accumulated in that region and are more toxic for the cancerous cells than the normal ones^7^.

If the chemotherapeutic conjugated nanoparticles have radio sensitization properties, their effects are improved. The best example of this type of drug delivery system is the gold nanoparticles (GNPs). GNPs attract the attentions due to their low toxicity, biocompatibility, and the ability to bind to the different types of the molecules^2,8^. Their utilization is safe and they have anti-angiogenic properties. GNPs are among the high-Z agents (Z = 79) that have a high X-ray absorption ability and when they enter into the body they increase the radiation sensitivity. Therefore, high energy form from the irradiation is concentrated inside the tumor, and increases the efficiency of the radiotherapy. Hence lowered doses of the radiation are used *in vivo*^2^.

GNPs have a high surface area that results in their high ability of binding to different molecules and having a high half-life in the body. Therefore, they attach with high payload to the chemotherapeutic drugs, decrease the requirements for administration of high doses of the drugs and decrease their toxic effects for non-cancerous cells in the body^9,10^.

Among the different methods of GNPs production, is biological technique^11^ which in comparison to the other types of nanoparticles production, is safe, inexpensive, fast and does not release any harmful and toxic byproducts in the environment^12^. In the biological technique of GNPs nanoparticles production, there are several bacterial and fungal strains that can produce this type of nanoparticles^13^. In this method the microbial proteins play the role as reducing and capping agents of the produced nanoparticles that made them stable even when they are in the close contacts^12,14,15^. In the conjugation process these proteins actively bind to the drugs without the requiring of any additional linkers. It seems that these proteins help the nanoparticles to enter into the cancerous cells^16^.

Due to the insufficient data about the assessment of the conjugation possibility of the chemotherapeutic drugs to the biologically produced GNPs, recent research has tried to analyze the conjugation of three different chemotherapeutic drugs, capecitabine, tamoxifen and paclitaxel, with two different physiological forms, liquids and tablets, to the biologically produced GNPs without the use of any additional linkers. The conjugation of the drugs to the GNPs was proved by different methods, and then their anti-cancer activity was evaluated against two different cancerous cell lines. The results from this basic study will be beneficial for the future studies.

## Materials and Methods

### GNPs biosynthesis

*Fusarium oxysporum* (PTCC 238-21-3) was purchased from the Pasteur Institute of Iran and used for extracellular GNPs production. The fungal strain was cultured in a flask containing 150 ml of Sabouraud’s dextrose broth (SDB) (Merck, Germany) in the incubator at 30 °C for 7 days. The obtained mycelia were separated using centrifugation process at 6000 rpm for 10 min and the fungal culture supernatant was used for the production of GNPs. For this aim, 100 μl of 1M HAuCl_4_ (SigmaAldrich, USA) solution was added to 100 ml of the fungal culture supernatant (the final concentration of gold ions was 1 mmol). The negative control flask that contained 100 ml of the sterile SDB plus 100 μl of 1M HAuCl_4_ was prepared. The flasks were incubated at 40 °C, 200 rpm, for 24 h. The first sign of GNPs formation was the color changing of the reaction mixture from yellow to dark purple or red^17,18^.

### Washing and sterilizing the GNPs

Because the GNPs in the fungal culture supernatant contained some impurities that may interfere with the conjugation process, the mixture was washed using ddH_2_O. In this regard, GNPs were centrifuged under 14,700 rpm for 30 min. The pellet was suspended in ddH_2_O and the above mentioned process was repeated twice. Finally, the pellet was suspended in ddH_2_O and sterilized using tyndallization method. Briefly, the suspension was heated indirectly under water steam for 30 min and incubated overnight at 37 °C. The process was repeated three times^15^.

### Characterization of the produced GNPs

#### Visible spectral analysis

If the GNPs are produced, they have maximum absorbance peak around the wavelength of 500–550 nm due to the surface plasmon resonance (SPR) of the nanoparticles. Therefore, the optical density (OD) of the color changed fungal culture supernatant was achieved using spectrophotometer. The used blank was ddH_2_O. The used wavelength ranges were from 450 to 650 nm^18,19^.

#### Transmission electron microscopy (TEM)

The size and shape of the produced GNPs were analyzed using Zeiss Leo 910 transmission electron microscope. For this aim, 5 μl of the color changed fungal culture supernatant was placed on a carbon coated copper grid and after 15 seconds the excess of the solution was removed and the grid was air dried and analyzed under TEM^18,20^.

#### X-ray diffraction (XRD) analysis

XRD was performed to confirm the presence of the elemental gold in the fungal culture supernatant. For this aim, the sample was freeze dried and used for XRD analysis. The diffracted intensities were obtained from 30° to 80° at 2°θ using a Philips automatic X-ray diffractometer^18,20^.

#### Fourier transform Infrared (FTIR) analysis

In order to determine the structure of the GNPs and prove the presence of capping proteins on the surfaces of the nanoparticles, FTIR analysis was applied. For this aim the sandwich method was performed and because the sample was in the aqueous form, the AgCl plates were used. A small drop of the sample was placed on one of the plates and the second plate was placed on the top. After achievement a thin layer of the sample, the plates were placed into the sample holder and FTIR spectrum of the GNPs was measured by the use of Nicolet FTIR instrument in the range of 500–4000 cm^−1 ^^10^.

#### Drugs preparation

Capecitabine (500 mg), tamoxifen (10 mg) and paclitaxel (6 mg/ml) drugs were purchased from OSVE, Iran hormone and Sobhan pharmaceutical companies, respectively. 100 mg of the tablet powder of capecitabine and tamoxifen were dissolved in 100 ml of ddH_2_O, separately^21^ and concentration of them were determined using UV-visible spectrophotometer. The standard curve obtained from each pure drug concentration versus OD used from the previous research articles. According to Harini *et al*. capecitabine has two maximum absorbance peaks at 308 and 616 nm^22^ and according to Albert *et al*. tamoxifen has one maximum absorbance peak at 236 nm^23^. Therefore for capecitabine 616 nm and for tamoxifen 236 nm wavelengths were used in the UV-visible spectrophotometer analysis, the used blank was ddH_2_O and the concentration of each drug was determined based on the obtained OD.

#### Synthesis of drug loaded GNPs

For drug loading experiment, 0.5 ml of each drug stock solution was added drop wise to 50 mL of GNPs solution in the sterile condition. The mixtures were maintained for 72 h at room temperature under stirring condition. In order to remove the unbound drugs from the solution, the mixtures were centrifuged at 15,000 rpm for 10 min and each pellet was suspended in ddH_2_O. The above mentioned procedure was repeated twice^10^.

### Characterization of drugs conjugated GNPs

#### Visible spectral analysis

The maximum absorbance peaks of the drugs conjugated GNPs after conjugation process were changed. Therefore, ODs of the drugs conjugated GNPs were achieved by the aid of the spectrophotometer. The utilized blank was ddH_2_O and the wavelength ranges were from 400 to 650 nm^24^.

#### Dynamic light scattering (DLS) analysis

The size distribution of the GNPs before and after conjugation with the tested drugs was obtained using the Zetasizer Nano range instrument (Malvern, UK). For this aim each drug conjugated solution and GNPs were sonicated for 60 min and analyzed at room temperature. The Z-Average (d.nm) and polydispersity index (PDI) of the solutions and the zeta potential of the GNPs were determined^25^.

#### Fourier Transform Infrared (FTIR) analysis

In order to determine conjugation of the drugs to the GNPs, FTIR analysis was employed. For this aim, FTIR spectra of the drugs conjugated GNPs were measured by the aid of Nicolet FTIR instrument in the range of 500–4000 cm^−1^. The non-conjugated drugs were utilized as the controls^24^. The samples were in the liquid form and their preparation method was described earlier.

### *In vitro* analysis

#### Cell culture

In order to analyze the drugs conjugated GNPs cytotoxicity, two different cancerous cell lines, AGS (ATCC CRL-1739) and MCF7 (ATCC HTB-22), were purchased from the Pasteur Institute of Iran. AGS cell line was sensitive to capecitabine^26^ and MCF7 cell line was sensitive to tamoxifen^27^ and paclitaxel^28^. For both of the cell lines Dulbecco’s Modified Eagle’s medium (DMEM, Sigma–Aldrich, USA) which was enriched with 10% fetal bovine serum (FBS, Sigma–Aldrich, USA) and 1% penicillin streptomycin solution (Sigma–Aldrich, USA) was used as a working medium.

#### Cytotoxicity assay

Before the cytotoxicity assay, GNPs and all the drugs conjugated GNPs were frieze-dried and the obtained powder of them were weighted. 1 mg of each powder was dissolved in 1 ml of the working medium (the concentration of each sample was 1 mg/ml). Eight of test samples: GNPs, each of the tested drugs conjugated GNPs and each of the tested drugs (with the concentration of 1 mg/ml in the working medium), were analyzed by methyl thiazol tetrazolium (MTT) assay that utilized 3-(4,5-dimethylthiazol-2-yl)-2,5-diphenyltetrazolium bromide dye (SigmaAldrich, USA) solution. For this aim, 8000 cells/well were seeded in each well of the 96 wells micro titer plate in the presence of the working medium and incubated at 37 °C for 24 h in the presence of 5% CO_2_. The obtained 80% monolayer cells achieved and then the medium was removed and the cell surfaces were washed by the use of phosphate buffered saline (PBS). Then, 200 μl of the working medium was added to all the wells. The experiments started from the second well of the second row of the plate. In order to avoid the evaporations, the margins of the plate were not used. In the plate that was seeded with AGS cell line, capecitabine, capecitabine conjugated GNPs and GNPs were tested. Each row of the plate was filled by one of the mentioned samples. In this regard, 200 μl (1 mg/ml) of the sample was added to the second well and the solution was mixed by the use of pipeting. Therefore the concentration of the sample in the second well was 0.5 mg/ml. Then 200 μl of it was transferred to the third one and the obtained concentration of the sample was 0.25 mg/ml. This procedure continued until the 10^th^ well and 200 μl of the 10^th^ well was discharged. Therefore the concentration of the sample in the 10^th^ well was 0.0019 mg/ml. The 11^th^ well was a control and contained 200 μl of the working medium. By this technique each sample was diluted by half. For the plates that were seeded with MCF7 cell line, two rows of the plate were loaded by tamoxifen, tamoxifen conjugated GNPs and two rows of the plate were loaded by paclitaxel and paclitaxel conjugated GNPs. One row was filled with GNPs alone. The tests were carried out in duplicates and all the plates were incubated at 37 °C for 24 h in the presence of 5% CO_2_. In the next day, all the wells of the palates except the margins wells were filled with 10 μl of 5 mg/ml MTT dye solution. The plates were incubated at 37 °C for 4 h in the presence of 5% CO_2_, the solution was removed and 100 μl of dimethyl sulfoxide (DMSO, Sigma Aldrich, USA) was added to all the wells. The plates were incubated for 20 min and the ODs of the wells were measured using ELISA reader at the wavelength of 570 nm^29,30^. Finally, the IC_50_ value for each sample was analyzed and compared with the obtained IC_50_ values from the others. The percentages of the viable cells were calculated from the obtained ODs^31^.

#### Inductively coupled plasma (ICP) measurements

In order to analyze the amounts of the GNPs and drug conjugated GNPs that entered into the used cell lines, ICP study was performed. For this aim, 10000 cells/well were seeded in a 6 wells micro titer plate. Two wells were seeded with AGS and three were seeded with MCF7 cell lines. The cells were incubated in the presence of the working medium at 37 °C for 24 h in the presence of 5% CO_2_. The obtained 80% monolayer cells achieved and then the medium was removed. For the wells that were seeded by AGS cell line, 700 μl of the working medium was added to each well and one well was filled with 700 μl (1 mg/ml) of capecitabine conjugated GNPs and the other one was filled with 700 μl (1 mg/ml) of GNPs. For the wells that were seeded by MCF7 cell line, 700 μl of the working medium was added to each well. One well was filled with 700 μl (1 mg/ml) of tamoxifen conjugated GNPs, the other one was filled with 700 μl (1 mg/ml) of paclitaxel conjugated GNPs and the last one was filled with 700 μl (1 mg/ml) of GNPs. The plate was incubated in the above mentioned condition for 3 h and the medium was discharged. The surfaces of the cells were washed trice using PBS and the cells were detached using trypsin-EDTA solution (Sigma Aldrich, USA). In order to quantify the concentrations of the gold in the cytoplasms of the tested cells ICP-OES 730-ES, Varian instrument was employed in the presence of the standard graph^31^.

#### Stability analysis

In order to analyze the stability of the drugs conjugated GNPs the samples were kept in the room temperature for 6 months. The *in vitro* stability of the drugs was analyzed by incubation the drugs conjugated GNPs (200 µl) with the equal amounts of the working medium at room temperature for 24 h and 2 weeks. The samples were analyzed using visible spectrophotometer. The utilized blank was ddH_2_O and the wavelength ranges were from 400 to 650 nm^24^.

## Results

### GNPs biosynthesis

**Figure 1:**
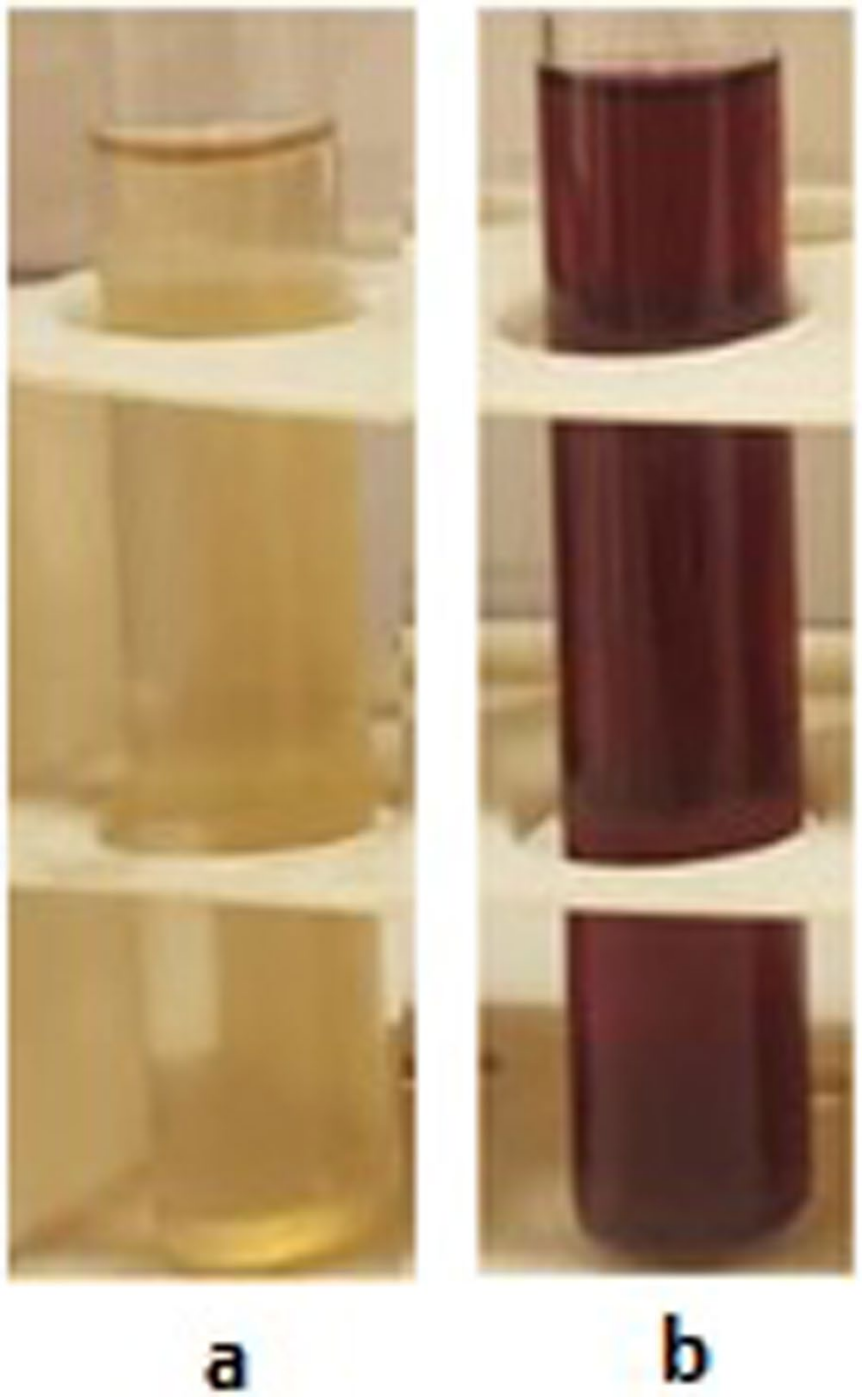
The obtained color changed supernatant due to the GNPs production. (**a**) Fungal culture supernatant before GNPs production and (**b**) Fungal culture supernatant after GNPs production.

The color of the fungal supernatant in contrast to the negative control, turned from yellow to red due to the SPR of the GNPs. Figure 1 illustrates the obtained color changed supernatant.

### Characterization of the produced GNPs

#### Visible spectral analysis

**Figure 2:**
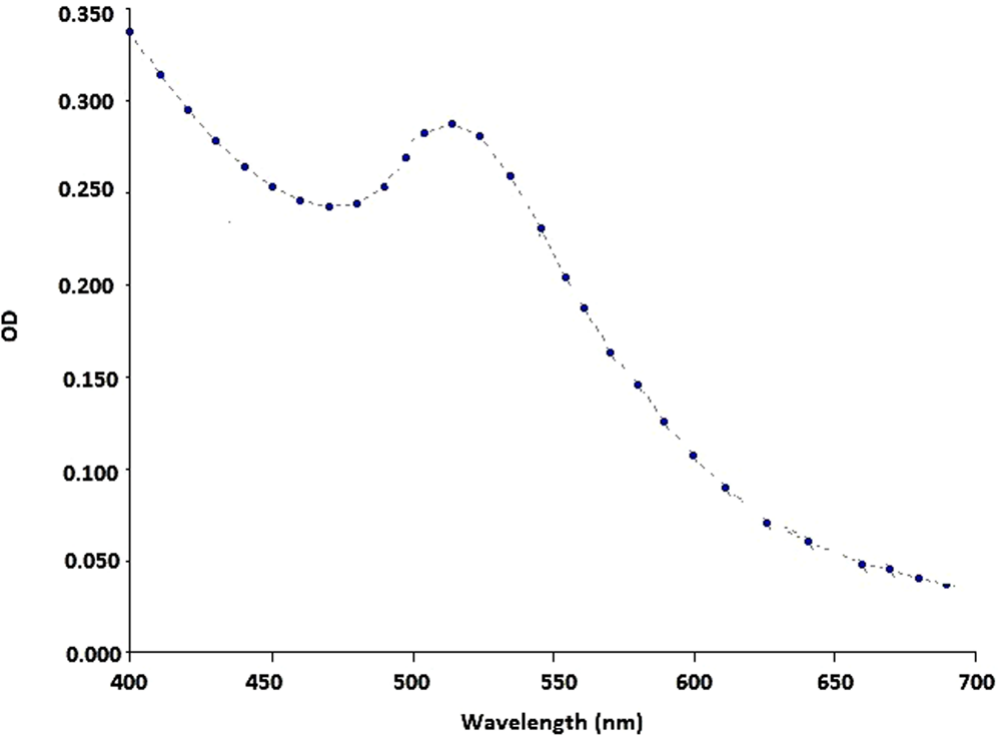
Obtained visible spectrum after GNPs production and dilution. The maximum absorbance is observed around 524 nm.

Visible spectral analysis result showed that the GNPs sample had maximum absorbance peak around 524 nm. Because the sample had high turbidity, it was diluted 1:15. Figure 2 indicates the obtained spectrum.

#### Transmission electron microscopy (TEM)

**Figure 3:**
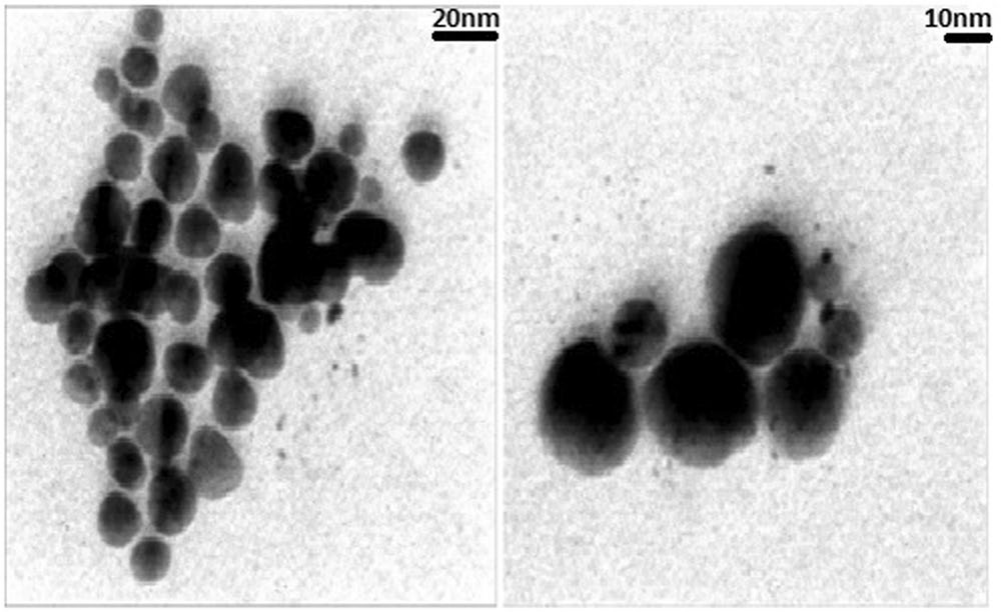
TEM results of the produced GNPs. The particles had average sizes less than 20 nm, with spherical and hexagonal shapes (scale bares = 20 and 10 nm).

The results revealed that the produced GNPs had spherical and hexagonal shapes with the average sizes less than 20 nm. Figure 3 demonstrates the obtained TEM images.

#### X-ray diffraction (XRD) analysis

**Figure 4:**
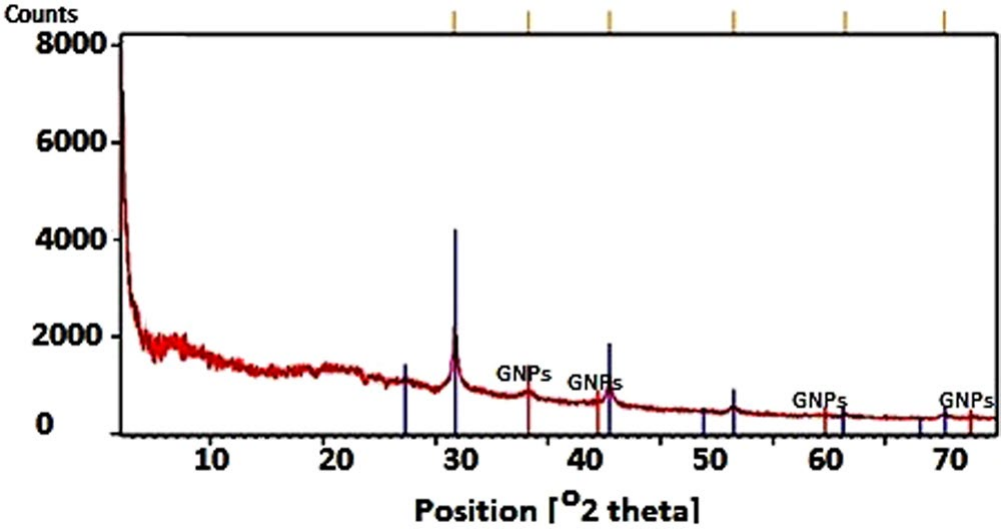
XRD spectrum obtained from the produced GNPs. The GNPs peaks are determined in this figure.

XRD results confirmed the presence of the elemental gold in the fungal culture supernatant. GNPs had cubic crystalline form with the calculated density of 19.28 g/cm^3^. Its reference code was 03-65-2870. Figure 4 depicts the obtained spectrum.

#### Fourier transform Infrared (FTIR) analysis

**Figure 5:**
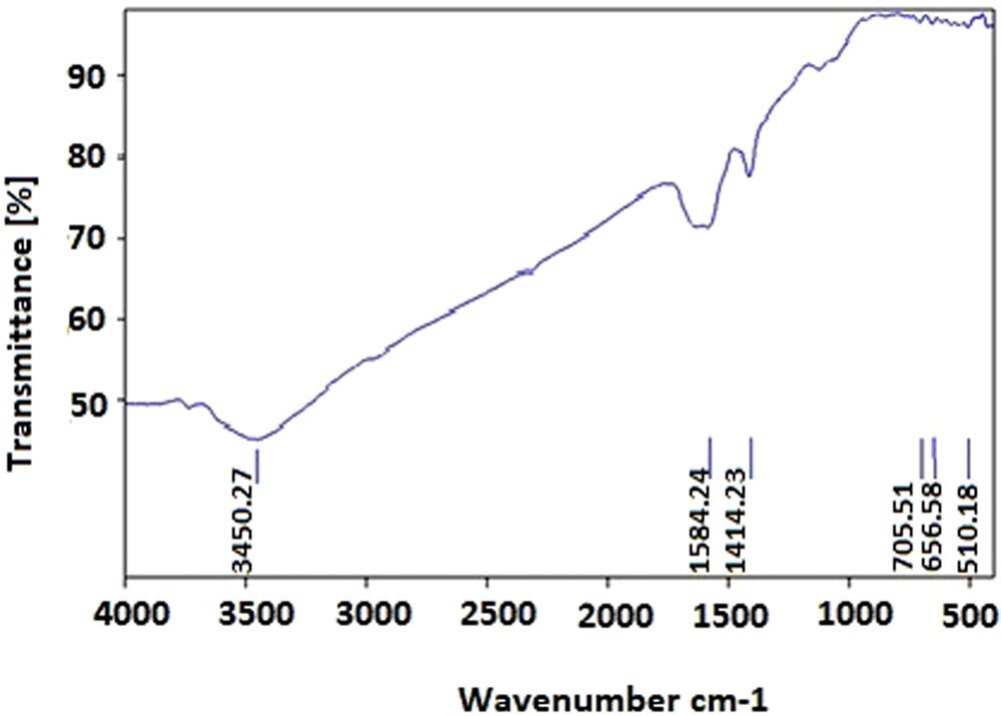
The obtained FTIR result of the GNPs. The peak positions are determined in this figure.

In order to determine the structure of the GNPs, FTIR analysis was used. The obtained results revealed that different types of proteins surrounded the GNPs which indicated the presence of capping agents on the surfaces of GNPs. The peak at wave number of 3400 cm^−1^ indicated the presence of OH group, and its wideness was due to the acidic OH group. The poor peaks presented in the ranges from 3500 to 3400 cm^−1^ were due to the stretching vibrations of NH bands that represented the presence of the amide bands of the protein. The spectrum available at wave number of 1500 cm^−1^ had two branches that one branch belonged to C=O, and its lateral peak belonged to the bending vibration of the NH group that represented the amide bands. The spectrum available at wave number of 1400 cm^−1^ represented the CH_2_ band. The peaks that observed below the wave number of 1000 cm^−1^ indicated the metallic bands. Figure 5, indicates the obtained FTIR result of the GNPs.

#### Drugs preparation

The concentration of the tablet form drugs was determined using UV-visible spectrophotometer. Based on the obtained ODs, the concentration of capecitabine was 54 μg/ml and for tamoxifen was 47 μg/ml.

### Characterization of drugs conjugated GNPs

#### Visible spectral analysis

**Figure 6:**
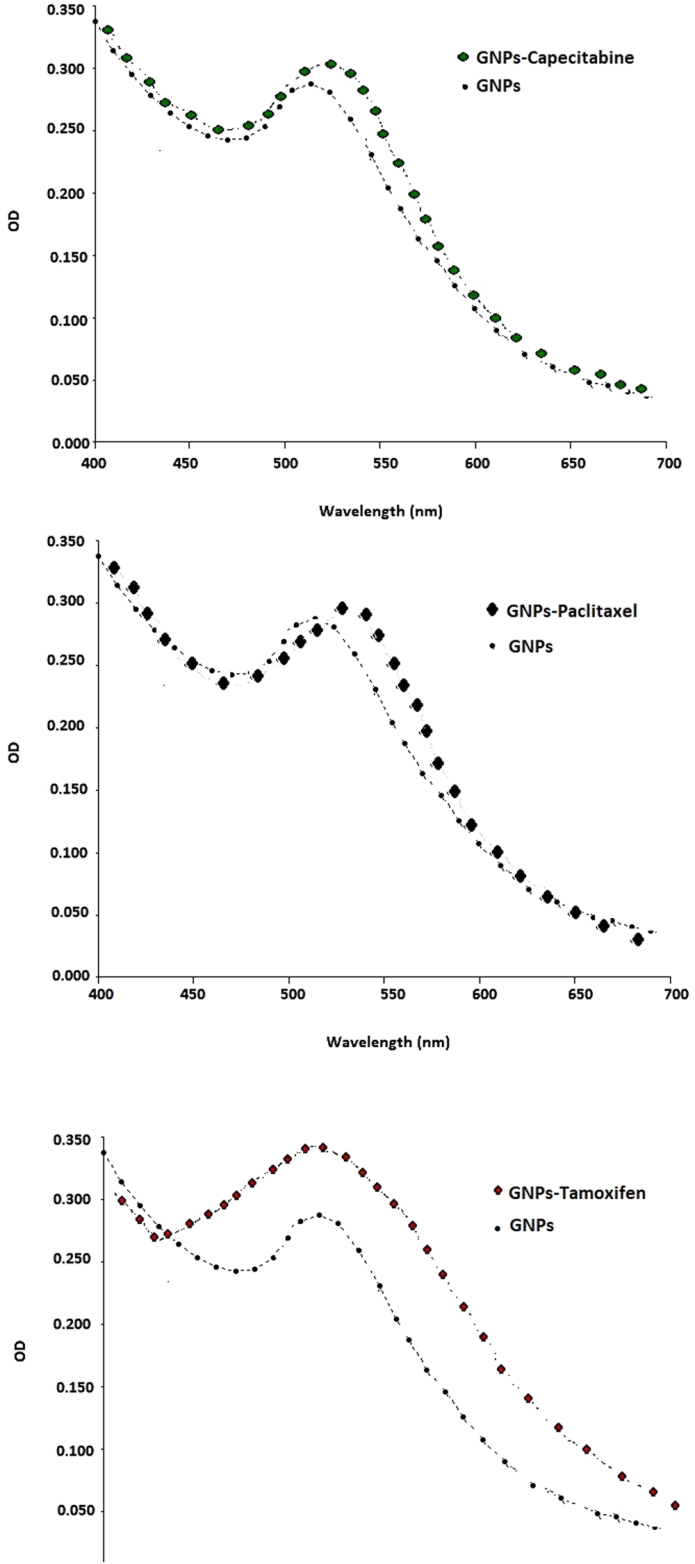
The obtained spectra and comparison between each drug conjugated GNPs and GNPs. The ODs of the GNPs after 72 h of the conjugation process were changed and their maximum absorbance peaks were shifted to higher wavelength.

Three different drugs (capecitabine, tamoxifen and paclitaxel) conjugated GNPs were analyzed using the spectrophotometer instrument. The results demonstrated that although the ODs of the GNPs after conjugation were altered and their maximum absorbance peaks were shifted to higher wavelengths, these maximum absorbance peaks were limited to 500–550 nm. Figure 6 illustrates the obtained spectra after 72 h of the conjugation process.

#### Dynamic light scattering (DLS) analysis

**Figure 7:**
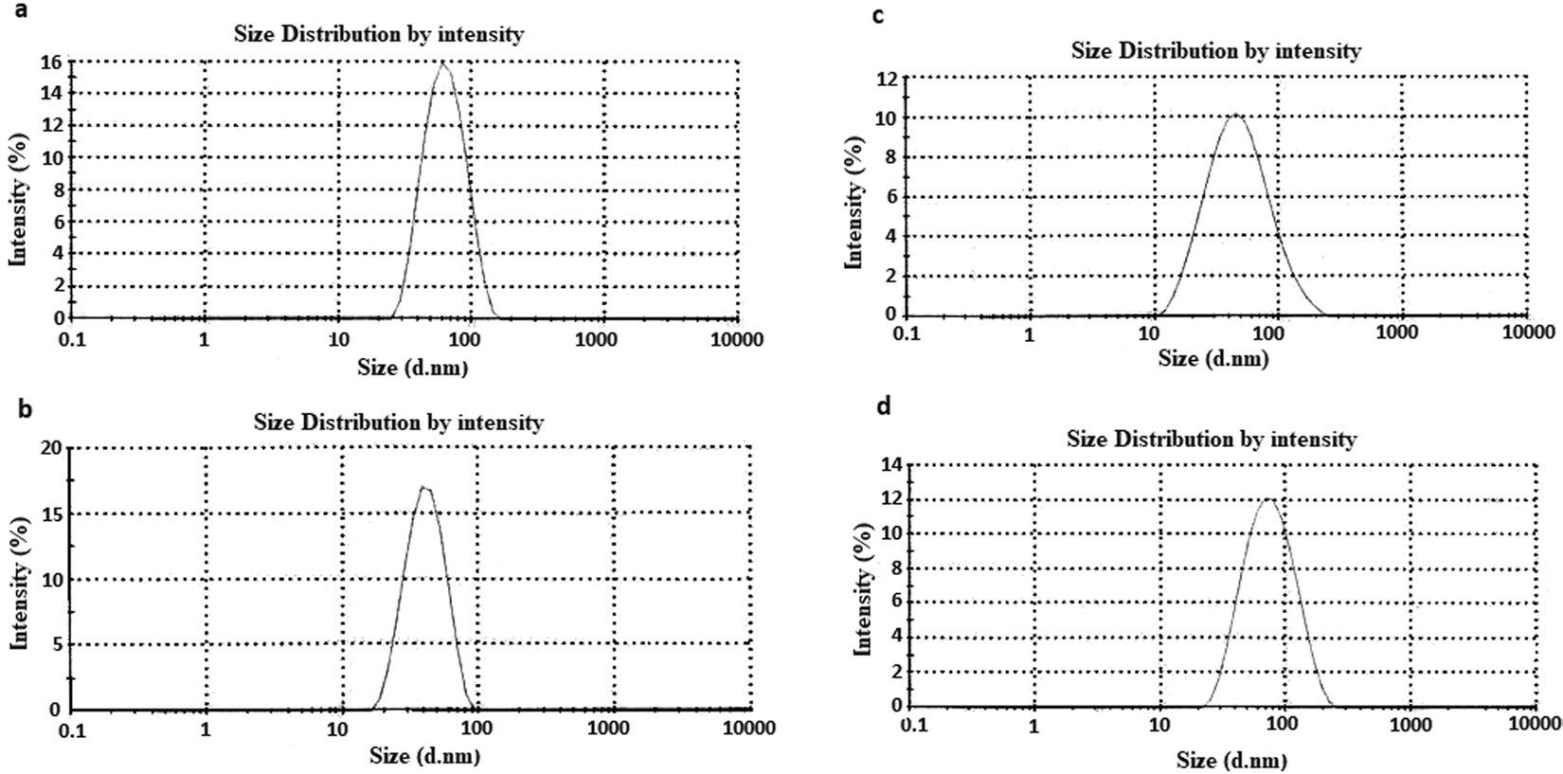
DLS results of the GNPs before and after conjugation process. (**a**) GNPs, (**b**) capecitabine conjugated GNPs, (**c**) tamoxifen conjugated GNPs and, (**d**) paclitaxel conjugated GNPs.

DLS results revealed that GNPs before and after conjugation process had different sizes and dispersities which confirmed the changes in the surface of the GNPs due to the GNPs-drugs core shell formation. Totally, as it can be seen in Fig. 7, the average size and PDI of the GNPs after conjugation with all the three tested drugs increased. The zeta potential of the GNPs was −46 ± 0.4 mV. Table 1, represented the obtained data.

**Table 1:** The obtained Z-Average (nm) and PDI for the four tested solutions (i.e. GNPs, capecitabine conjugated GNPs, tamoxifen conjugated GNPs and paclitaxel conjugated GNPs).

The tested solution	Z-Average (nm)	PDI
GNPs	54.27 ± 3.1	0.165 ± 0.37
Capecitabine conjugated GNPs	83.51 ± 5.5	0.141 ± 0.12
Tamoxifen conjugated GNPs	61.91 ± 2.1	0.196 ± 0.22
Paclitaxel conjugated GNPs	61.37 ± 5.1	0.211 ± 0.40

#### Fourier transform infrared (FTIR) analysis

**Figure 8:**
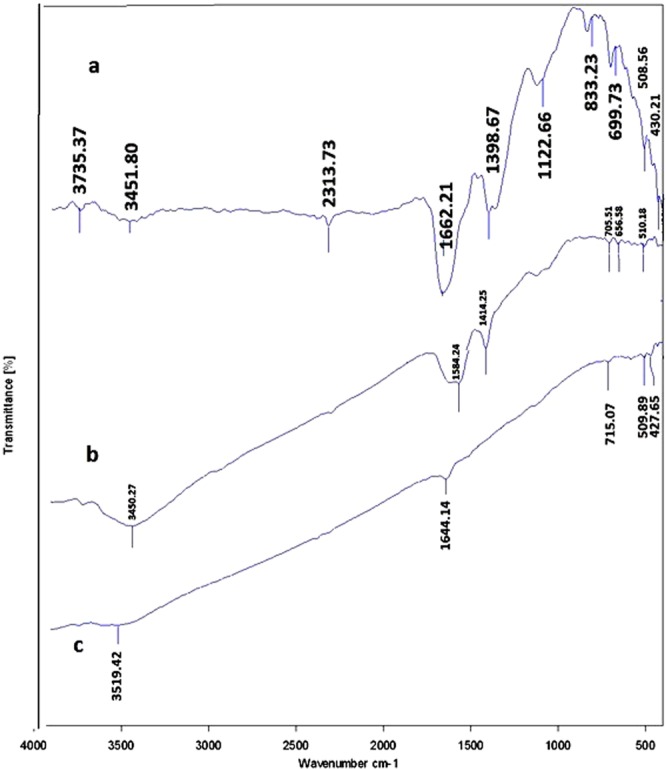
The obtained FTIR spectra for capecitabine and capecitabine conjugated GNPs. (**a**) Capecitabine conjugated GNPs. (**b**) GNPs and C. capecitabine.

**Figure 9:**
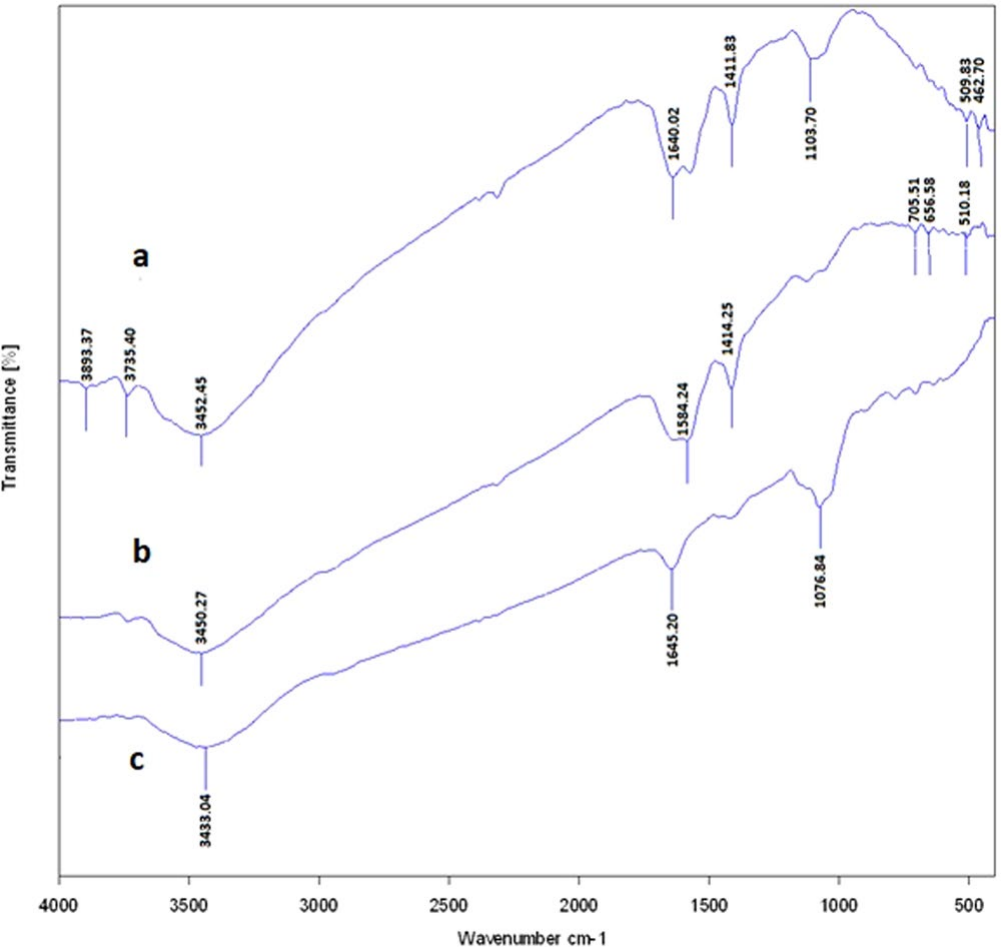
The obtained FTIR spectra for tamoxifen and tamoxifen conjugated GNPs. (**a**) Tamoxifen conjugated GNPs. (**b**) GNPs and C. tamoxifen.

**Figure 10:**
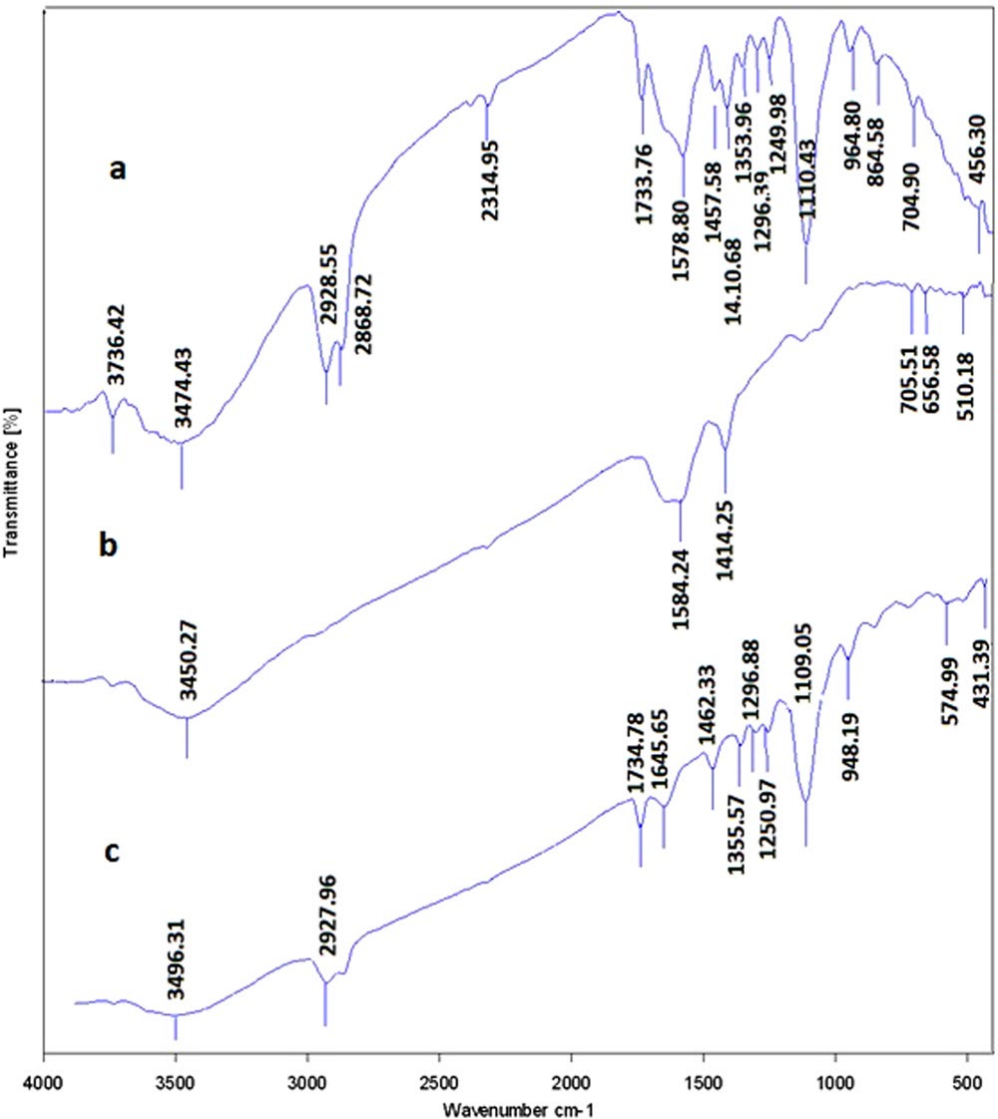
The obtained FTIR spectra for paclitaxel and paclitaxel conjugated GNPs. (**a**) Paclitaxel conjugated GNPs. (**b**) GNPs and C. paclitaxel.

As it was described earlier, in order to prove the conjugation of the drugs to the GNPs, each of the drug and the drug conjugated GNPs was analyzed by the use of FTIR. Figures 8–10 represent the obtained results from three different drugs and their conjugates. As Fig. 8 illustrates, for capecitabine, the peak at wave number of 3400 cm^−1^ indicated the presence of OH of the alcoholic and phenolic groups. The peak at wave number of 1600 cm^−1^ belonged to C=O that its location shifted due to its presence in the ring and its placement next to the nitrogen atom. The peaks of the nitrogen bands observed in this area. The peaks below the wave number of 1000 cm^−1^ related to the different groups that presented on the ring. For capecitabine conjugated GNPs, all the peaks of the drug (at wave number of 3400 cm^−1^ belonged to phenolic group, at wave number of 1600 cm^−1^ belonged to carbonyl group and peaks below the wave number of 1000 cm^1^ belonged to the different groups on the ring) observed in the spectrum. At wave number of 1600 cm^−1^ the carbonyl group of the drug shifted down due to its placement near the nitrogen of the capping proteins. In addition, all the peaks related to the nanoparticles such as the peak at wave number of 3400 cm^−1^ which belonged to OH group indicated in the obtained FTIR results, which demonstrated that the GNPs without any fractures bound with the drug and formed core shells.

In case of tamoxifen as demonstrated in Fig. 9, the peak at wave number of 3400 cm^−1^ indicated the presence of OH of the alcoholic and phenolic groups. The peak at wave number of 1600 cm^−1^ belonged to C=O that its location shifted due to its presence in the ring and its placement next to the nitrogen atom. The peaks of the nitrogen bands observed in this area. The peaks in the ranges from the wave numbers of 1000 to 1100 cm^−1^ belonged to the C–O bands. For tamoxifen conjugated GNPs, the peaks of the metallic bands presented. Also the peaks in the ranges from wave numbers of 1000 to 1100 cm^−1^ that belonged to the drug C–O bands displayed. It is possible to prove the presence of the drug with the peak area at wave number of 1600 cm^−1^ that belonged to C=O and nitrogen bands (it confirmed the presence of the types I and II amides). The peaks in the ranges at wave numbers of 1600 to 3700 cm^−1^ belonged to the carbonyl and amide II groups that were in the GNPs which indicated that the GNPs without any fractures bound with the drug and formed core shells.

For paclitaxel, as indicated in Fig. 10, the peaks at wave numbers of 1000, 1300 and 1700 cm^−1^ indicated the presence of ester bands which were in the formulation of the drug. The width peak at 3400 cm^−1^ indicated the presence of OH group of the alcoholic and phenolic structures. This peak was weak due to the formation of hydrogenic bands with the other drug components. The peaks below the wave number of 1000 cm^−1^ (especially the one at wave number of 900 cm^−1^) and the ones around wave number of 1600 cm^−1^, demonstrated the positions of the substitutions on the aromatic rings. The powerful peaks that observed at wave number of 1600 cm^−1^ were attributed to C=C bands of the aromatic rings. The spectrum available at wave number of 2900 cm^−1^ had two branches that illustrated the SP^3^ hybrids in CH bands. For paclitaxel conjugated GNPs, in the obtained spectrum all the paclitaxel peaks specially the ones that were seen at wave numbers of 900 and 1600 cm^−1^ showed. Also the drug ester, CH and C=C bands were observed. The peak at 3500 cm^−1^ which belonged to the amide II group of the GNPs was more visible. The carbonyl peak of the GNPs also demonstrated, resulting that the drug bound to the GNPs and formed core shells.

### *In vitro* analysis

#### Cytotoxicity assay

**Figure 11:**
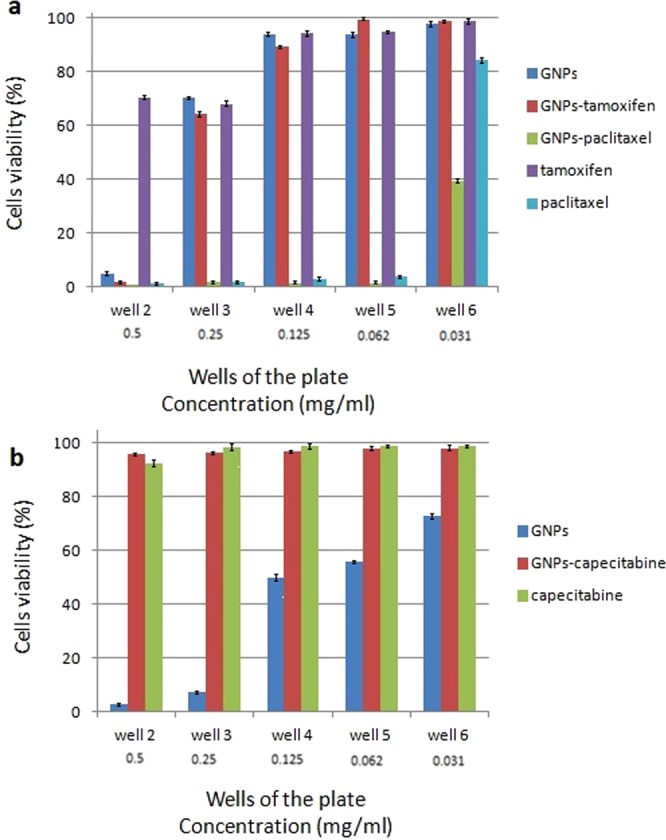
The MTT assay obtained results for MCF7 and AGS cell lines. Only the second from 6^th^ wells (with the concentration of 0.5 to 0.031 mg/ml) are represented. 11^th^ well was the positive control and the percentage of cell viability was 100%. (**a**) The obtained results for MCF7 cell line and (**b**) the obtained results for AGS cell line.

In order to analyze the cytotoxicity of each drug conjugated GNPs and each of the drugs, two different cancerous cell lines, AGS and MCF7, were employed and the tests were carried out in duplicates. As it was mentioned previously, the analyses started from the second well of the plate. MTT assay revealed different results for each group of the specific drug conjugated GNPs, GNPs and the used drug. The results are indicated in Fig. 11. MTT assay result for capecitabine in the AGS cell line totally illustrated that the drug was deactivated in the cell culture. No toxic effects were observed for the cells that were treated with different doses of capecitabine and capecitabine conjugated GNPs. GNPs had dose dependent toxic effects in the cell culture and its IC_50_ was in the 4^th^ well with the concentration of 0.125 mg/ml. Also for tamoxifen in the MCF7 cell line, the drug was deactivated and the cells could grow from the second well of the plate. For GNPs and tamoxifen conjugated GNPs their IC_50s_ were equal and were in the 3^th^ well with the concentration of 0.25 mg/ml which indicated the toxicity of the tamoxifen conjugated GNPs was due to the toxicity of the GNPs and not the drug.

Paclitaxel and paclitaxel conjugated GNPs in the MCF7 cell line showed toxic effects in the cell culture that the IC_50_ value for paclitaxel was calculated in the 6^th^ well with the concentration of 0.031 mg/ml and for paclitaxel conjugated GNPs was in the 7^th^ well with the concentration of 0.0156 mg/ml. Paclitaxel conjugated GNPs induced more toxic effects than the drug and GNPs alone. Finally, GNPs induced toxic effects in both of the used cell lines but more toxic effects in the AGS cell line was observed than the MCF7 one. Figure 11a,b represent the MTT assay results and compare them with each other for MCF7 and AGS cell lines, respectively. Although the tests were carried out in duplicates, the obtained results were the same.

#### Inductively coupled plasma (ICP) measurements

**Figure 12:**
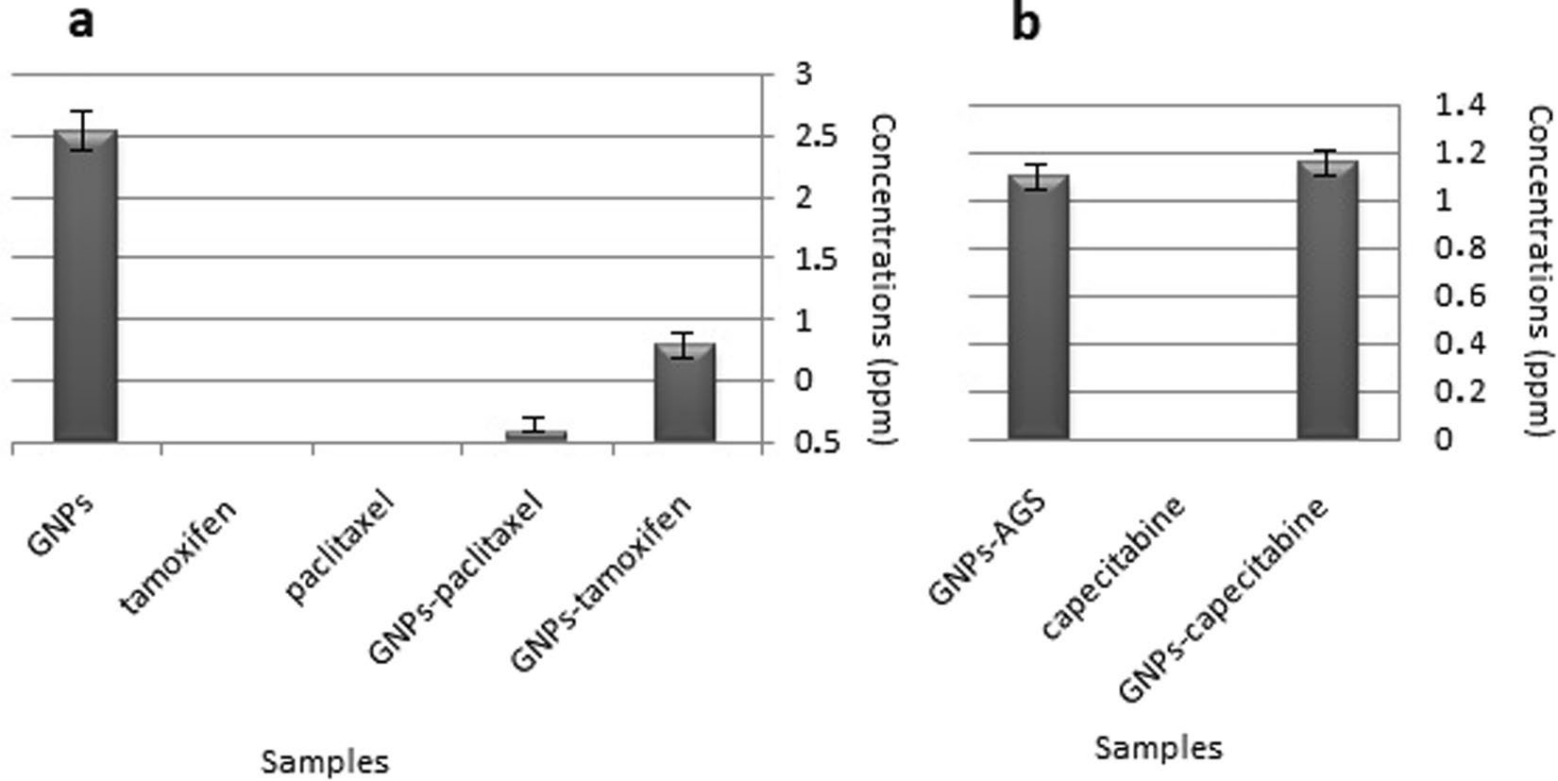
The concentrations (ppm) of elemental gold resulted from ICP measurement for three different used drugs conjugated GNPs, drugs and GNPs alone in two different cell lines. (**a**) MCF7 cell line and (**b**) AGS cell line.

In order to analyze the amounts of the GNPs that entered into the used cell lines, ICP study was performed. As it was mentioned in the materials and methods section, all the wells of the plate was filled with 700 μl of 1 mg/ml of each sample. Therefore both of the cell lines received the same concentration of each tested sample. The results indicated that GNPs could penetrate to the AGS and MCF7 cell lines but the amounts of them in the cells were different. The amount of the GNPs that entered into the AGS cell line was 1.1 ppm and into the MCF7 cell line was 2.53 ppm. The amounts of the capecitabine conjugated GNPs, tamoxifen conjugated GNPs, and paclitaxel conjugated GNPs in the used cell lines were 1.15, 0.77 and 0.07 ppm, respectively. Therefore, the ability of the GNPs in the same concentration in entering to different cells after conjugation with the drugs was different. Figure 12a,b show and compare the obtained data for MCF7 and AGS cell lines, respectively.

#### Stability analysis

The results of the stability test of the drugs conjugated GNPs after 6 months of the experiments showed that the obtained maximum absorbance peaks had small variations which confirmed the drugs conjugated GNPs were stable at room temperature. Furthermore the *in vitro* stability analysis of the drugs conjugated GNPs showed the same results. Therefore the GNPs after conjugation with the drugs had high stability in the environmental and *in vitro* conditions. The obtained maximum absorbance peaks for GNPs and drugs conjugated GNPs are shown in Table 2.

**Table 2:** The obtained maximum absorbance peaks for GNPs and drugs conjugated GNPs after 72 h and 6 months in the environmental and after 24 h and 2 weeks in the *in vitro* conditions.

Samples	Maximum absorbance peak (nm)			
	Environmental condition		*In vitro* condition	
	72 h	6 months	24 h	2 weeks
GNPs	524	529	520	525
Capecitabine conjugated GNPs	533	539	533	531
Tamoxifen conjugated GNPs	525	528	528	524
Paclitaxel conjugated GNPs	545	548	547	545

## Discussion

Capecitabine, tamoxifen and paclitaxel are three different chemotherapeutic drugs that are utilized for the treatment of different cancers. Capecitabine is used for the treatment of gastric, gastroesophageal, esophageal and colorectal cancers^32^. Tamoxifen is used for the treatment of early and advanced breast cancer^33^ and paclitaxel is used for the treatment of metastatic breast, leucopenia, ovarian, liver, prostate, lung and neck cancers^34^. It is important to note that the tested cell lines in the current study were susceptible to the used drugs. Based on the literature review, AGC cell line was susceptible to capecitabine^26^ and MCF7 cell line was susceptible to both of the tamoxifen^27^ and paclitaxel^28^ drugs.

In the chemotherapy procedure, the use of these drugs had some disadvantages such as the appearance of resistance cancerous cells and their undesirable side effects in the patient’s body which may be the main reason of the patient’s death. In order to overcome these disadvantages, there are some solutions such as application of different drug delivery agents that purposefully target the tumor cells and conjugation of the chemotherapeutic drugs to some especial types of nanoparticles^2^. The second method is acknowledged to be inexpensive, so the recent research attempted to use GNPs for conjugation with the above mentioned drugs. By this method, drugs were conjugated to the GNPs with high affinity and therefore, the doses of the used drugs in the chemotherapy procedure were decreased. It means that the side and toxic effects of the drugs were lowered^2,35^.

Chemical, physical and biological methods are three main techniques of nanoparticles production^12,14,15,20,30^. Recently, due to the toxicity of the nanoparticles that are produced by chemical and physical techniques, the biological method of nanoparticles production is under more considerations. In the biological technique, different types of bacterial and fungal strains produce the nanoparticles actively in or out of the microbial cells which depends on the placement of the cell’s enzymes and active component^36^. Some microorganisms have the ability of the production of both types of nanoparticles. A fungal strain, *F. oxysporum* is among them. It was indicated that this nonpathogenic fungus has a high ability of production of different types of nanoaprticles due to its powerful secretion systems and is used widely in the field of nanoaprticles production^19^. In the nanoparticles production procedure the fungal proteins and enzymes play a role in reducing and capping the produced nanoparticles that made them stable even when they are in the close contacts^19^. GNPs are among the different nanoparticles that are produced by this fungal strain. The biologically produced GNPs are acknowledged as the most biocompatible type of nanoparticles that induce low toxic effects in the cell culture^19^. They have a high surface area and high ability for binding to different types of bio-molecules. The binding of the bio- molecules to GNPs are directly accomplished without requiring any additional foreign linkers due to the presence of the capping proteins on the surfaces of the produced GNPs^16^. There is a paucity of research about the conjugation ability of the chemotherapeutic drugs to the biologically produced GNPs. Kumar *et al*. have used resveratrol as the anticancer agent for conjugation with the GNPs that were produced by a bacterial strain and found that the drug conjugated GNPs acted better in the cell culture than the drug alone^24^. Therefore recent research attempted to analyze the conjugation of three different chemotherapeutic drugs, capecitabine, tamoxifen and paclitaxel, with two different physiological forms, liquids and tablets, to the biologically produced GNPs without the use of any additional linkers.

In the first part of the experiments, the fungal strain was cultured in SDB and after the separation of the mycelia; the fungal culture supernatant was employed for the extracellular production of GNPs. It was indicated that in the extracellular production of nanoparticles some fungal proteins, enzymes, polysaccharides and other secreted substances are responsible for nanoparticles production^37^. The extraction of nanoparticles that are produced out of the cells is easier than the ones that are produced in the cells^37^; therefore, in this study the extracellular production way was selected. After the production of GNPs, their presence was confirmed by the aid of spectrophotometer, TEM, XRD and FTIR analyses. In the spectrophotometer analysis, GNPs had maximum absorbance peak around 524 nm due to their SPR. If the nanoparticles form clusters, the maximum absorbance peak shifts to the higher wavelength or this peak is not be observed^38^. Therefore, this test indicated that the GNPs were produced. TEM and XRD results proved the presence of round nanoparticles with the average sizes less than 20 nm that they had at least four distinctive peaks in the XRD resulted spectrum. DLS results revealed that the Z-Average of the monodisperse nanoparticles was 54.27 ± 3.1 (Table 1). Therefore the results of the DLS analysis were different from the TEM results because the TEM images were taken from the one part of the TEM grid but DLS analysis revealed the average sizes of the total solution. The DLS results have higher accuracy than the TEM ones.

Totally, FTIR analysis revealed the presence of different peaks at different wave numbers corresponded to the metallic bands of the gold and amide bands and functional groups of the proteins that all together proved the presence of the proteins on the surfaces of the GNPs which acted as the capping agents.

For the synthesis of the drug loaded GNPs, the drugs were added to the washed GNPs and the mixtures were maintained for 72 h at room temperature under stirring condition. Because two of the used drugs were in the tablet form, firstly, they were dissolved in ddH_2_O. Unfortunately, these drugs displayed no toxic effects in the cell culture which may be due to their physical forms. Because in this study conjugation process was slow it is assumed that the drugs were deactivated due to their low half-lives. Also it was indicated that these tablet forms have low water solubility and they should be dissolved in methanol^39^. Methanol itself had toxic effects and could not be used in the cell culture so it was not utilized, however, in a research carried out by Dreaden *et al*. the chemically produced GNPs were utilized for the conjugation with tamoxifen and demonstrated that the drug conjugated GNPs actively killed the breast cancerous cells; they have used the pure form of the drug that was dissolved in methanol and the purification was done using gel filtration method. Also they have utilized the additional linkers for the preparation of tamoxifen conjugated GNPs^35^.

Due to these and the other probably unknown reasons, these two types of the drugs could not induce desirable toxic effects in the tested cell cultures. It was indicated previously that the use of other forms of the drugs with high stability, cold incubation of the drugs during the conjugation process, and low conjugation time resulted in proper and desirable conjugates. Therefore, it is very significant to use the drugs with high stability in the environmental conditions. The third drug which was in the liquid form was paclitaxel. As the results revealed, this drug had high stability and therefore it acted in the cell culture and induced toxic effects. Heo *et al*., assessed the paclitaxel conjugation with the chemically produced GNPs in order to increase its anticancer properties. They have used different ligands such as biotin for purposefully cancer treatment. Their study had a desirable result but unlike the present study, GNPs were prepared by the aid of chemical technique and their surfaces were functionalized using biotin^40^.

In order to analyze the conjugation of the drugs to the GNPs, spectrophotometer, DLS and FTIR analyses were performed. Spectrophotometer analysis revealed that the ODs of the GNPs after conjugation with the drugs were shifted to higher wavelengths but as it has indicated in the results section, the obtained maximum absorbance peaks were limited to 500–550 nm which confirms that the GNPs after conjugation with the drugs remained in their nano size. Also after 6 months of the first experiment these maximum absorbance peaks showed small variations which confirmed that the drugs conjugated GNPs were stable at room temperature. These conjugates were stable in the *in vitro* condition too which means that adsorption of the serum proteins on the surfaces of the GNPs did not affect on the nature of the GNPs.

As it was mentioned previously based on the obtained DLS data, although bigger sizes of GNPs after conjugation to the drugs and formation of core shell were achieved (i.e. the increase in Z-Average results), their dispersities were acceptable and this factor was not changed drastically.

The FTIR results revealed that all the three types of the drugs were successfully conjugated to the biologically produced GNPs. Totally, the FTIR spectrum that was obtained for each drug conjugated GNPs illustrated the peaks of the GNPs and the ones with the especial used drug that indicated the successful conjugation of the drugs to the GNPs. Therefore, it can be concluded that three used drugs with different chemical compositions and different half lives, even if they are deactivated, can conjugate to the GNPs which is due to the high binding affinity of GNPs to different types of molecules.

In order to remove the unbound drugs from the solution, the conjugates were washed. By this process the unbound drugs could not affect the results of the cytotoxicity assay^24^. Before the MTT assay, each sample (i.e. drug conjugated GNPs and GNPs) was freeze-dried, weighed and with the concentration of 1 mg/ml was used for MTT assay. Also the drug powder was weighted and used with the same concentration. Although all the samples were used in the same concentrations, the weight of each drug in the drug conjugated GNPs sample was lower than the drug sample alone. This is because of the removal the unbound drugs and also presence the weight of the GNPs in the conjugated samples. Although the exact amount of each drug in the drug conjugated GNPs sample was lower than the non conjugated form of the corresponded drug but in the case of paclitaxel the drug conjugated GNPs had better anticancer activity than the drug sample. It is recommended to calculate the exact amount of the unbound drugs after washing the conjugates and use the equal amount of it that is banded to the GNPs in the drug tested group.

As it was mentioned previously, in the case of MTT assay each set of the drug, drug conjugated GNPs and GNPs indicated different results. The GNPs in both of the AGS and MCF7 cell lines showed toxic effects that their toxicity was higher for AGS than the MCF7 cell lines. Their IC_50s_ were 0.125 mg/ml and 0.25 mg/ml, respectively. This may be due to the inherent differences between these two different cell lines. In the assessment of the capecitabine and capecitabine conjugated GNPs, the results expressed that both of them were deactivated in the cell culture. Although GNPs induced some toxic effects in the cell culture, after conjugation with the drug they did not induce any toxicity in the AGS cell line. Probably, the GNPs functional toxic groups were covered with the used drug. This was coincidence with the increase of the Z-Average of GNPs after conjugation with capecitabine (83.51 ± 5.5 nm). ICP results illustrated that GNPs in both of the conjugated and non conjugated forms could penetrate into the cells, therefore, if the drug was active, it would induced high toxicity in the cell culture because the nanoparticles could pass the drugs from the cell surface to the cytoplasm.

Tomuleasa et al., used cisplatin, doxorubicin, and capecitabine conjugated GNPs and expressed that GNPs increased the susceptibility of cancerous cells to the used conjugated drugs. Despite the present study, the chemically produced GNPs in addition to L-Aspartate as a chemical linker were used. In the previous study, capecitabine did not lose its activity due to the use of low incubation time (about 1 h) for conjugation to the GNPs.

In the assessment of tamoxifen and tamoxifen conjugated GNPs, unlike the capecitabine tested group, after conjugation of the drug to the GNPs, the functional groups of the GNPs did not fill and therefore, GNPs in the conjugated and non conjugated forms could induce the toxic effect in the MCF7 cell line (IC50s = 0.25 mg/ml). ICP results showed that GNPs in both of the conjugated and non conjugated forms could penetrate into the cells but in contrast to the conjugated form, the penetration of its non conjugated form was better. This may be due to the attachment of the drugs on the surfaces of the nanoparticles, and formation of core shells with bigger sizes which affects its penetration into the cells. Again, this was coincidence with the increase of the Z-Average of GNPs after conjugation with tamoxifen (61.91 ± 2.1 nm). As the spectrophotometer data revealed, although for all the three used drugs conjugated GNPs the maximum absorbance peaks were shifted to higher wave numbers, but the peaks were limited between 500–550 nm which was an indication of remaining of the GNPs in their nano sizes even after conjugation with the drugs^24,38^. Moreover, once again, because of the nature of the tamoxifen, like the capecitabine, the drug was deactivated.

In the paclitaxel tested group, the drug preserved its stability and both of the drug and drug conjugated GNPs indicated toxic effects in the cell culture. As it is observed in the Fig. 11, the drug conjugated GNPs induced more toxic effect in contrast to the drug alone. Their IC_50s_ were 0.0.0156 mg/ml and 0.031 mg/ml, respectively. ICP measurement revealed that both of the GNPs and drug conjugated GNPs could penetrate into the MCF7 cell line but the penetration of the drug conjugated GNPs was lower than the GNPs alone. The DLS results confirmed this hypothesis because the Z-Average and PDI of the GNPs after conjugation with paclitaxel increased. Therefore, the drug conjugated GNPs with lower penetration than the GNPs could induce higher toxic effects and its toxic effect was not due to the toxicity of GNPs alone.

Recent research has tried to analyze the conjugation possibility of three different chemotherapeutic drugs to the biologically produced GNPs without the use of any additional linkers. The production of the biologically produced GNPs is easy, safe, eco friendly and fast. By this method some microbial secretions such as proteins act as the reducing and capping agents. In our study the capping agents that their presence on the surfaces of GNPs was confirmed by FTIR method, acted as the linker which leaded to one step conjugation of the drugs to the GNPs. Therefore in comparison with the other studies^2,23,40^ that have used chemical linkers which banded to the drugs, the one step and easy linking of the functional groups of the drugs to the capping agents of GNPs is one of the most important advantages of this method. One of the disadvantages of the present study was the time that was spent for conjugation process (for all the drugs was 72 h). This linking time was not optimized and it needs more assessment to lowering the conjugation time.

In future, it is recommended to use especial ligands in conjugation with GNPs that are specific for the receptors of the utilized cancerous cell lines. It is recommended to decrease the time and use cold incubation for conjugation process and to use the other types of stable drugs.

## Conclusions

In conclusion, the biologically produced GNPs had the ability of conjugation with different types of chemotherapeutic drugs but it is very important to choose the drugs which preserve their activity in the linking process. Our findings revealed that although all the three used drugs could conjugate to the GNPs, the drugs which were in the tablet form were non toxic in the cell culture. This was due to their deactivation and instability after dissolving in ddH_2_O. The results from this paper showed that bigger sizes of GNPs after conjugation with different drugs achieved which affected on their cellular penetration. Although the conjugated form of the drug-GNPs had bigger sizes and lower penetration to the cells but they could induce better anti cancer activity than the non conjugated drugs. By the use of the biologically produced GNPs, the conjugation method was easy and inexpensive without the use of any additional linkers. Results from the current study suggest that the paclitaxel conjugated GNPs may has therapeutic potential for treatment of the breast or other types of cancers.

In future, it is recommended to use especial ligands and drugs in conjugation with GNPs that are specific for the receptors of the used cancerous cell line.

## References

[1] Parkin DM, Pisani P, Ferlay J (1999). Global cancer statistics. CA cancer j cli.

[2] Davidi ES (2018). Cisplatin-conjugated gold nanoparticles as a theranostic agent for head and neck cancer. Head & neck.

[3] Yvon A-MC, Wadsworth P, Jordan MA (1999). Taxol suppresses dynamics of individual microtubules in living human tumor cells. Molecular biology of the cell.

[4] Agüeros M, Zabaleta V, Espuelas S, Campanero M, Irache J (2010). Increased oral bioavailability of paclitaxel by its encapsulation through complex formation with cyclodextrins in poly (anhydride) nanoparticles. J. Control. Release.

[5] Ghosh P, Han G, De M, Kim CK, Rotello VM (2008). Gold nanoparticles in delivery applications. Adv. Drug Deliv. Rev.

[6] Ferrari M (2005). Cancer nanotechnology: opportunities and challenges. Nat. Rev. Cancer.

[7] Liu X, Braun GB, Qin M, Ruoslahti E, Sugahara KN (2017). *In vivo* cation exchange in quantum dots for tumor-specific imaging. Nat Commun.

[8] Popovtzer A (2016). Actively targeted gold nanoparticles as novel radiosensitizer agents: an *in vivo* head and neck cancer model. Nanoscale.

[9] Han G, Ghosh P, Rotello VM (2007). Functionalized gold nanoparticles for drug delivery. Nanomedicine.

[10] Dhar S, Reddy EM, Shiras A, Pokharkar V, Prasad BELEV (2008). Natural gum reduced/stabilized gold nanoparticles for drug delivery formulations. Chem.: Eur. J.

[11] Yahyaei B, Arabzadeh S, Pourali P (2014). An alternative method for biological production of silver and gold nanoparticles. JPAM.

[12] Pourali P (2014). Impregnation of the bacterial cellulose membrane with biologically produced silver nanoparticles. Curr Microbiol.

[13] Pourali P (2012). Biological synthesis of silver and gold nanoparticles by bacteria in different temperatures (37 C and 50 C). J Pure. Appl Microbiol.

[14] Nikbakht M, Yahyaei B, Pourali P (2015). Green synthesis, characterization and antibacterial activity of silver nanoparticles using fruit aqueous and methanolic extracts of Berberis vulgaris and Ziziphus vulgaris. J Pure. Appl Microbiol.

[15] Pourali P, Yahyaei B (2016). Biological production of silver nanoparticles by soil isolated bacteria and preliminary study of their cytotoxicity and cutaneous wound healing efficiency in rat. Journal of Trace Elements in Medicine and Biology.

[16] Pourali P, Yahyaei B, Afsharnezhad S (2018). Bio-Synthesis of Gold Nanoparticles by *Fusarium oxysporum* and Assessment of Their Conjugation Possibility with Two Types of β-Lactam Antibiotics without Any Additional Linkers. Microbiology.

[17] Rezaei A, Pourali P, Yahyaei B (2016). Assessment of the cytotoxicity of gold nanoparticles produced by *Bacillus cereus* on hepatocyte and fibroblast cell lines. Iranian Journal of Cellular and Molecular Researches.

[18] Yahyaei B, Nouri M, Bakherad S, Hassani M, Pourali P (2019). Effects of biologically produced gold nanoparticles: toxicity assessment in different rat organs after intraperitoneal injection. AMB Express.

[19] Pourali P (2017). Biosynthesis of gold nanoparticles by two bacterial and fungal strains, *Bacillus cereus* and *Fusarium oxysporum*, and assessment and comparison of their nanotoxicity *in vitro* by direct and indirect assays. Electron J Biotechn.

[20] Pourali P (2013). The effect of temperature on antibacterial activity of biosynthesized silver nanoparticles. Biometals.

[21] Tomuleasa C (2012). Gold nanoparticles conjugated with cisplatin/doxorubicin/capecitabine lower the chemoresistance of hepatocellular carcinoma-derived cancer cells. J Gastrointestin Liver Dis.

[22] Harini U, Pawar A (2016). Validated UV and visible spectrophotometric method for the estimation of capecitabine–A cancer drug. Der Pharmacia Lettre.

[23] Albert EL, Shirosaki Y, Abdullah CAC (2018). Drug Release and Kinetic Study of Tamoxifen Citrate conjugated with Magnetite Nanoparticle for Drug Delivery Application. IJAER.

[24] Kumar CG, Poornachandra Y, Mamidyala SK (2014). Green synthesis of bacterial gold nanoparticles conjugated to resveratrol as delivery vehicles. Colloids Surf. B.

[25] Ruparelia JP, Chatterjee AK, Duttagupta SP, Mukherji S (2008). Strain specificity in antimicrobial activity of silver and copper nanoparticles. Acta biomaterialia.

[26] Zhu H (2013). The synergistic effects of low dose fluorouracil and TRAIL on TRAIL-resistant human gastric adenocarcinoma AGS cells. Biomed Res Int.

[27] Zheng A, Kallio A, Harkonen P (2007). Tamoxifen-induced rapid death of MCF-7 breast cancer cells is mediated via extracellularly signal-regulated kinase signaling and can be abrogated by estrogen. Endocrinology.

[28] Zuo KQ, Zhang XP, Zou J, Li D, Lv ZW (2010). Establishment of a paclitaxel resistant human breast cancer cell strain (MCF-7/Taxol) and intracellular paclitaxel binding protein analysis. J Int Med Res.

[29] Pourali P, Yahyaei B (2018). Effect of Silver Nanoparticles Produced by Paenibacillus on Rat Cutaneous Wound Healing. Journal of Mazandaran University of Medical Sciences.

[30] Pourali P, Razavian Zadeh N, Yahyaei B (2016). Silver nanoparticles production by two soil isolated bacteria, *Bacillus thuringiensis* and *Enterobacter cloacae*, and assessment of their cytotoxicity and wound healing effect in rats. Wound Rep Reg.

[31] Murawala P, Tirmale A, Shiras A, Prasad B (2014). *In situ* synthesized BSA capped gold nanoparticles: effective carrier of anticancer drug methotrexate to MCF-7 breast cancer cells. Mater. Sci. Eng. C.

[32] Ajani J (2006). Review of capecitabine as oral treatment of gastric, gastroesophageal, and esophageal cancers. Cancer.

[33] Clemons M, Danson S, Howell A (2002). Tamoxifen (‘Nolvadex’): a review: Antitumour treatment. Cancer Treat Rev.

[34] Priyadarshini K, Keerthi AU (2012). Paclitaxel against cancer: a short review. Med chem.

[35] Dreaden EC, Mwakwari SC, Sodji QH, Oyelere AK, El-Sayed MA (2009). Tamoxifen− poly (ethylene glycol)− thiol gold nanoparticle conjugates: enhanced potency and selective delivery for breast cancer treatment. Bioconjugate Chem.

[36] Yahyaei, B., Manafi, S., Fahimi, B., Arabzadeh, S. & Pourali, P. Production of electrospun polyvinyl alcohol/microbial synthesized silver nanoparticles scaffold for the treatment of fungating wounds. *Appl Nanosci* 1–10 (2018).

[37] Yahyaei B (2016). Production, assessment, and impregnation of hyaluronic acid with silver nanoparticles that were produced by *Streptococcus pyogenes* for tissue engineering applications. APPL BIOL CHEM.

[38] Alkilany AM, Murphy CJ (2010). Toxicity and cellular uptake of gold nanoparticles: what we have learned so far?. J Nanopart Res.

[39] Simultaneous spectroscopic estimation and validation of gemcitabine and tamoxifen in synthetic mixture by first order derivative method. *IJPSR* **6**(9), 4000–4003 (2015).

[40] Heo DN (2012). Gold nanoparticles surface-functionalized with paclitaxel drug and biotin receptor as theranostic agents for cancer therapy. Biomaterials.

